# State–Space Forecasting of *Schistosoma haematobium* Time-Series in Niono, Mali

**DOI:** 10.1371/journal.pntd.0000276

**Published:** 2008-08-13

**Authors:** Daniel C. Medina, Sally E. Findley, Seydou Doumbia

**Affiliations:** 1 College of Physicians & Surgeons, Columbia University, New York, New York, United States of America; 2 Malaria Research & Training Center, FMPOS, Université de Bamako, Mali; 3 Mailman School of Public Health (Department of Population and Family Health), Columbia University, New York, New York, United States of America; The University of Queensland, Australia

## Abstract

**Background:**

Much of the developing world, particularly sub-Saharan Africa, exhibits high levels of morbidity and mortality associated with infectious diseases. The incidence of *Schistosoma* sp.—which are neglected tropical diseases exposing and infecting more than 500 and 200 million individuals in 77 countries, respectively—is rising because of 1) numerous irrigation and hydro-electric projects, 2) steady shifts from nomadic to sedentary existence, and 3) ineffective control programs. Notwithstanding the colossal scope of these parasitic infections, less than 0.5% of *Schistosoma* sp. investigations have attempted to predict their spatial and or temporal distributions. Undoubtedly, public health programs in developing countries could benefit from parsimonious forecasting and early warning systems to enhance management of these parasitic diseases.

**Methodology/Principal Findings:**

In this longitudinal retrospective (01/1996–06/2004) investigation, the *Schistosoma haematobium* time-series for the district of Niono, Mali, was fitted with general-purpose exponential smoothing methods to generate contemporaneous *on-line* forecasts. These methods, which are encapsulated within a state–space framework, accommodate seasonal and inter-annual time-series fluctuations. Mean absolute percentage error values were *circa* 25% for 1- to 5-month horizon forecasts.

**Conclusions/Significance:**

The exponential smoothing state–space framework employed herein produced reasonably accurate forecasts for this time-series, which reflects the incidence of *S. haematobium*–induced terminal hematuria. It obliquely captured prior non-linear interactions between disease dynamics and exogenous covariates (e.g., climate, irrigation, and public health interventions), thus obviating the need for more complex forecasting methods in the district of Niono, Mali. Therefore, this framework could assist with managing and assessing *S. haematobium* transmission and intervention impact, respectively, in this district and potentially elsewhere in the Sahel.

## Author Summary

Adequate forecasting and early warning systems are based upon observations of human behavior, population, disease time-series, climate, environment, and/or a combination thereof, whichever option best compromises among realism, feasibility, robustness, and parsimony. Fully automatic and user-friendly state–space forecasting frameworks, incorporating myriad options (e.g., expert opinion, univariate, multivariate, and spatial-temporal), could considerably enhance disease control and hazard mitigation efforts in regions where vulnerability to neglected tropical diseases is pervasive and statistical expertise is scarce. The operational simplicity, generality, and flexibility of state–space frameworks, encapsulating multiple methods, could conveniently allow for 1) unsupervised model selection without disease-specific methodological tailoring, 2) *on-line* adaptation to disease time-series fluctuations, and 3) automatic switches between distinct forecasting methods as new time-series perturbations dictate. In this investigation, a univariate state–space framework with the aforementioned properties was successfully applied to the *Schistosoma haematobium* time-series for the district of Niono, Mali, to automatically generate contemporaneous *on-line* forecasts and hence, providing a basis for local re-organization and strengthening public health programs in this and potentially other Sahelian districts.

## Introduction

Prevalent parasitic infectious diseases frequently evade the public health radar because infected individuals present with a clinical history that is characterized by a highly heterogeneous symptomatology. *Schistosoma* sp., also known as bilharzias, expose and infect more than 500 and 200 million individuals in 77 countries, respectively [1],[2]; however, only those with severe symptoms seek available treatment. Though sub-clinical *Schistosoma* sp. infection detrimentally impacts the health of infected individuals, the enormous impact of seemingly asymptomatic and mildly symptomatic infection remains difficult to quantify. Furthermore, *Schistosoma* sp. incidence continues to rise because of 1) numerous irrigation and hydro-electric projects, 2) steady shifts from nomadic to sedentary existence, and 3) ineffective control programs unable to cope with population growth. With the mounting evidence that *Schistosoma* sp. impose an enormous burden on, as well as their control have paramount importance to improve public health in, developing countries, intervention programs therein could benefit from parsimonious forecasting and early warning systems to enhance management and hazard mitigation of these parasitic infections [1]–[8].

Most individuals at risk of *Schistosoma* sp. infection reside between latitudes 36° N and 34° S where average fresh water temperatures range from 25 to 30° C [1], placing African states among the most affected countries. *Schistosoma mansoni* and *Schistosoma haematobium* account for most *Schistosoma* sp. infection in Africa [1],^2^. *S. mansoni* and *S. haematobium* cercarias enter the human circulation trans-cutaneously. Subsequently, adult forms mate, migrate, and lay eggs, which eventually lodge in the intestine (*S. mansoni*) or bladder (*S. haematobium*). Excreted eggs hatch under favorable aquatic conditions, releasing miracidia, which penetrate the intermediate snail host—in Africa, *S. mansoni* and *S. haematobium* infect *Biomphalaria* sp. and *Bulinus* sp. snails [8],[9]. Finally, mature cercarias emerge from their intermediate host to seek human reservoirs thus, perpetuating their life cycle [1]. Individuals infected with *S. mansoni* are usually asymptomatic or mildly symptomatic (rash, fever, aching, cough, diarrhea, and or gland enlargement). In serious infection, lodged *S. mansoni* eggs trigger a granulomatous immune response that may cause colonic obstruction, hemorrhages, portal hypertension, ascites, and life-threatening esophageal varicose. *S. haematobium* produces similar unspecific symptoms whereas its fully symptomatic form manifests primarily as terminal hematuria.

**Figure 1:**
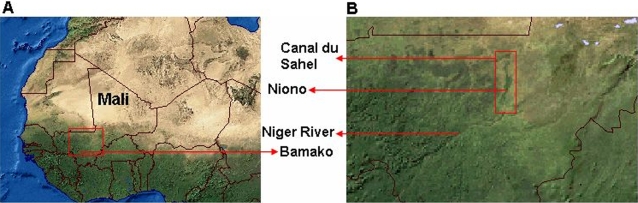
Satellite image of West Africa. Panel A: the Sahara desert and the savannah occupy the northern and southern West African landscapes, respectively, while the Sahel spans the intermediate fringe zone—Mali is transected by all three landscapes. Panel B corresponds approximately to an enlargement of the red demarcation in Panel A. The black line on the top of this panel delineates the southeastern Mauritanian border; the depicted segment of the Niger River flows in the southwest-northeast direction; the district of Niono, which is located 330 km northwest of Bamako and 100 km north of the Niger River along the *Canal du Sahel* (Segou Region), is situated within the red rectangle. This satellite image places the district of Niono in the Sahelian zone: poverty is extensive in the northern (semi-desert) and central (irrigated) regions; contrarily, poverty diminishes southward (near savannah areas) where mixed crops prevail. *Image source*: adapted with permission from Globalis, http://globalis.gvu.unu.edu (08/2007) [11].

Moreau *et al.* [10] reported the pervasive endemicity of *S. haematobium* in West Africa, particularly in the Sahel (**Figure 1**)—i.e. the sub-Saharan region that spans the entire east-west African axis, bordering the Sahara desert to the north and the Savanna to the south [11]. Conversely, his collaboration demonstrated that the prevalence of *S. mansoni* is greater in Sudanese and Guinean savannas [10]. Along this line of investigation, several epidemiological studies have evaluated the *Schistosoma* sp. prevalence in Mali [12]–[15], which ranks among the poorest countries in the world, and which is transected by savannas, the Sahel, and the Sahara desert.

**Figure 2:**
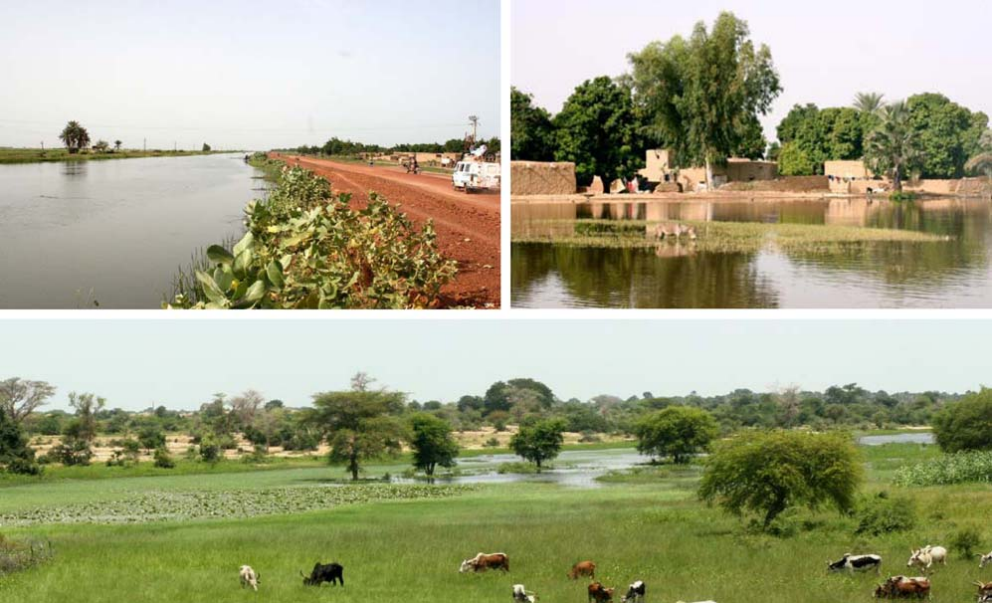
Irrigation system and stagnant water reservoirs in the district of Niono, Mali. This composite panel depicts irrigation canals (which support mainly rice monoculture) and stagnant water reservoirs where *Schistosoma haematobium* transmission may occur. District communities not only ingest water from the irrigation system but also wash their belongings, bathe, excrete, and amuse themselves in the canals, considerably increasing exposure to *S. haematobium*. Furthermore, rainfall precipitation fluctuations prompt the local authority (*Office du Niger*) to adjust irrigation management accordingly; for example, the *Office du Niger* may relax water control amid increased precipitation to better irrigate drier areas whilst collaterally enhancing water-flow through typically well-served agricultural fields—*S. haematobium* transmission suitability might then simultaneously increase and decrease in the former and latter scenarios, respectively.

Traore *et al.* [12] reported a 55% overall *S. haematobium* prevalence, with a case distribution orbiting the 7–14 age-category, in the district of Niono (Segou Region) and Dogon Plateau, Mali; *circa* 50 and 30% of infected individuals presented with clinical symptoms and pathologic lesions, respectively. The surveys conducted by Keita *et al.* [13] demonstrated that the *Schistosoma* sp. prevalence (7–14 age-category) in the community health center (CSCOM) service area of Molodo, in the district of Niono, was 72, 68, and 51% for *S. haematobium*, *S. mansoni*, and co-infection, respectively. Finally, Medina *et al.* [11] reported that *S. haematobium* is the 5^th^ most frequently diagnosed infectious disease, accounting for 2.5% of total CSCOM consultations in the district of Niono. The high prevalence of *Schistosoma* sp. in this district may be attributed to an extensive irrigation system that supports predominantly rice monoculture. Unfortunately, district communities not only ingest water from the irrigation scheme but also wash their belongings, bathe, excrete, and amuse themselves in the canals (**Figure 2**), considerably increasing exposure to *Schistosoma* sp. infection.

Notwithstanding the colossal scope of these parasitic infections in developing countries, only *circa* 0.5% of *Schistosoma* sp. investigations have attempted to predict their spatial and or temporal transmission distributions e.g. [1], [2], [16]–[18]—meriting special attention, Yang *et al.* [18] modeled both the spatial and temporal *S. japonicum* transmission dimensions in Jiangsu province, China. [The number of reports investigating *Schistosoma* sp. spatial and or temporal distributions roughly obtain *via* keyword-searching “schistosomiasis”, “*Schistosoma*”, “bilharzias”, “forecast”, “forecasting”, “prediction”, and keyword combinations at www.pubmed.com (09/25/2007). A meta-analysis is beyond the scope of this manuscript.] Regrettably, *S. haematobium* time-series (TS) forecasts are practically inexistent for Sahelian locations, such as Mali, where this neglected tropical disease tremendously deteriorate public health. Thus, the quest for robust *S. haematobium* TS forecasting methods to assist with preventing transmission, rapidly treating patients, as well as monitoring intervention impact must not be ignored.

**Figure 3:**
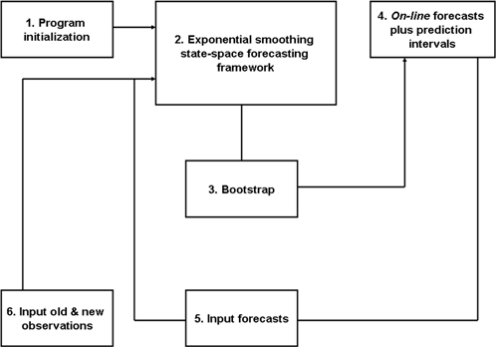
*On-line* forecast flow-chart. (1) Prior time-series (TS) observations initialize (2) the program that selects the best-performing exponential smoothing (ES) method within the state-space forecasting (ETS) framework, according to Equations 2 & 3 (*Methods*) as well as the Akaike's Information Criterion (*AIC*). Then, (3) Equations 2 & 3 simulate *h*-month horizon forecast path distributions with the best-performing ES method *via B* = 1000 ordinary residual bootstraps. (4) Mean forecast and 95% prediction interval (PI) values obtain as described in the *Methods* section. Subsequently, (5) the 1-month horizon forecast plus (6) the available TS (including the most contemporaneous observation) is supplied to (2, 3) the execution program to (4) revise forecasts and their 95% PI values. The automatic supply of contemporaneous TS observations into (2–6) yields revised *on-line* forecasts, i.e. external predictions. Basically, contemporaneous forecasts obtain *via* TS extrapolation whereby previous deviations between forecasts and their corresponding observations are exponentially adjusted with smoothing control values. For example, (1) the *Schistosoma haematobium* TS observations from January 1996 to December 1998 for the district of Niono, Mali, initialize (2–4) the ETS execution program that predicts consultation rates for January 1999 to May 1999 (assuming a 5-month horizon forecast). Once (5) the January 1999 forecast plus (6) the available TS (including the most contemporaneous observation of January 1999) become available to the *on-line* system, (2–4) the execution program cycles again and optimizes all considered ES methods, selecting the best-performing one (which may or may not be the same one employed prior to the arrival of this new observation). As a result, revised consultation rate predictions for February 1999 to June 1999 become available. This process repeats ceaselessly. This diagram was adapted from Medina *et al.* [11].

In this longitudinal retrospective (01/1996–06/2004) investigation, the *S. haematobium* consultation rate TS for the district of Niono, Mali (Fig. 1), was fitted with general-purpose exponential smoothing (ES) methods—encapsulated within a state-space framework—to produce contemporaneous *on-line* forecasts. *On-line* forecasts imply that historical records are continuously supplied to the execution program, which automatically revises external predictions. Although this state-space framework ignores direct effects from climate, public health intervention, and irrigation on *S. haematobium* TS evolution, it accommodates seasonal as well as inter-annual TS fluctuations. The ES methods within this framework may capture prior non-linear interactions between disease dynamics and the aforementioned covariates, potentially obviating the need for more complex predictive approaches in the district of Niono, Mali. [An intuitive overview of this ES state-space framework is conveyed by **Figure 3**.] Therefore, not only does this analysis address the paucity of reported *S. haematobium* TS investigations but it also demonstrates that this state-space framework could assist with managing *S. haematobium* infection in this district and possibly elsewhere in the Sahel.

## Methods

### Study setting

This longitudinal retrospective (01/1996–06/2004) *S. haematobium* TS investigation was conducted in the district of Niono, Mali (Fig. 1). Panel A in Fig. 1 is a satellite image that portrays Mali, with a projected population of 12 million in 2004 [19], along with its neighboring West African countries. Panel B—which corresponds approximately to an enlargement of the red demarcation in panel A—depicts the district of Niono (red rectangle), 330 km northwest of Bamako, 100 km north of the Niger River, in the Segou region. This district is a model location to test *S. haematobium* TS forecasting and early warning systems feasibility because its extensive irrigation network pervasively exposes communities to this neglected parasitic infection. Furthermore, the district of Niono shares epidemiological similarities with other regions in the Sahel where poverty- and disease-induced morbidity and mortality are rampant.

### Data pre-processing

The review of monthly clinical consultation records from the district of Niono, Mali, is part of a larger study on climate and health (“Putting climate in the service of public health”) that was approved by the “Columbia University Medical Center Institutional Review Board” (New York, U.S.A.) and the “Ethics Committee of the Mali National Medical School” (Bamako, Mali). Patient privacy was protected from inadvertent (or deliberate) violations because consultation records reflect monthly summaries that lack information with which individuals may be identified [11]. The assembled monthly data set (01/1996–06/2004) comprises consultation records for 20 diseases, which were tabulated by gender and age categories, from 17 CSCOM service areas within the district of Niono [11],[19],[20]. However, only the *S. haematobium* TS was analyzed here—diarrhea, acute respiratory infection of the lower tract (ARI), and malaria TS forecasts, as well as preliminary frequency description of all 20 diseases, have already been reported [11]. Of note, *Schistosoma* sp. consultation records reported by Medina *et al.* [11] and analyzed herein reflect cases of *S. haematobium*–induced terminal hematuria in over 99% of consultations, as discussed later, for which a single dose of 40 mg/kg of prazinquatel was prescribed in most cases.

Monthly *S. haematobium* consultation records for the 17 CSCOM service areas, both genders, and all ages were amalgamated. Rather than interpolating missing observations with imputed CSCOM-specific monthly median values and excluding ineligible CSCOM service area TS [11], this amalgamated consultation rate TS, {*y_t_*}, was estimated by simultaneous adjustment of time-dependent nominator (cases) and denominator (population) observations, according to Equation 1
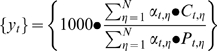
(1)where *C_t,η_* is the monthly number of CSCOM-specific *S. haematobium*-induced terminal hematuria consultations for both genders and all ages; *P_t,η_* is the time-dependent population of each CSCOM service area, which was adjusted for a national annual population growth rate of 3.2% [11],[19],[20]; *α_t,η_* = 0 if *C_t,η_* is missing for month *t* and CSCOM service area *η*, otherwise *α_t,η_* = 1; last, *N* = 17 is the total number of CSCOM service areas. The approximately random distribution of missing observations (∼17%) across months, years, and CSCOM service areas [11] ensures approximately unbiased {*y_t_*} estimation with Eq. 1, which holds as long as the denominator summation is positive. In simpler words, Eq. 1 estimates the monthly consultation rate for *S. haematobium*-induced terminal hematuria from CSCOM service areas for which records are available. Consultation rates and their forecasts are expressed as the monthly number of newly diagnosed *S. haematobium*–induced terminal hematuria cases *per* 1000 individuals in the district of Niono. Additional record details appear in **Table 1**, which was adapted from Medina *et al.* [11].

**Table 1:** Demographic and consultation record descriptions for the district of Niono, Mali.

CSCOM	Population (2004)	Time-series period	Missing dates	Missing months	% missing
**Boh**	7105	01/1996–06/2004	-	0	0.00
**Bolibana**	18321	01/1996–06/2004	1997	12	0.76
**Cocody**	6021	01/1996–06/2004	-	0	0.00
**Debougou**	25603	01/1996–06/2004	1997, 1998 (3)	15	0.94
**Diabaly**	16974	01/1996–06/2004	1997	12	0.76
**Diakiwere**	12269	01/1996–06/2004	1999 (3), 2003 (3)	6	0.38
**Dogofry**	24172	01/1996–06/2004	1997, 1998 (1)	13	0.82
**Fassoun**	5837	01/1996–12/1999	1997, 1999 (9)	21	1.33
**Kourouma**	8186	01/1996–06/2004	1997, 2001	24	1.52
**Molodo**	18379	01/1996–06/2004	1997, 2003 (6)	18	1.14
**Nampala**	7972	01/1996–06/2004	1996 (4), 1997, 1999 (9)	25	1.58
**Nara**	24161	01/2000–06/2004	2000, 2001, 2002, 2003 (6)	42	2.65
**Pogo**	11893	01/1996–06/2004	1997, 2003 (3)	15	0.94
**Siribala**	22745	01/1996–06/2004	1997, 2001 (3)	15	0.94
**Sokolo**	14672	01/1996–06/2004	1997, 1999 (3)	15	0.94
**Werekela**	14431	01/1996–06/2004	1996, 1997	24	1.52
**Niono**	40000	01/2000–06/2004	2000, 2001 (2), 2002 (1)	16	1.01
**Total**	278741	1584 months	-	272	17.2

The total projected (2004) population in the district of Niono, Mali, is 278 741 individuals, inhabiting approximately 20 000 km^2^. The projected number of individuals served by each community health center (CSCOM) service area within this district is tabulated under the *Population* heading. The population from each CSCOM service area was adjusted with the national annual population growth rate (3.2%) before the *Schistosoma haematobium* consultation rate time-series (TS) was calculated with Equation 1 (*Methods*) [19],[20]. Potential records are listed under *Time-series period*. Unavailable CSCOM service area records appear under *Missing dates*—the number of missing monthly records for each year is listed in parenthesis otherwise records for the whole year are missing. These are totaled under *Missing months* and expressed as percentages from the total number of possible records (across all CSCOM service areas and years) under the *% missing* heading. Of note, the Niono CSCOM service area, which includes the district center and immediate periphery, is one of the 17 CSCOM service areas within the district of Niono, Mali. This table was adapted from Medina *et al.* [11].

### Time-series forecasts

The amalgamated TS was fitted with ES methods, which are encapsulated within a state-space framework hereafter referred to as ETS for error (E), trend (T), and seasonal (S) components. The E component is either additive (A) or multiplicative (M); T and S components may be A, M, or inexistent (N); last, T may also be dampened additively (A_d_) or multiplicatively (M_d_). For example, the ETS method MMN has E(M), T(M), and S(N) structures. Therefore, there are 30 possible ES combinations within this forecasting framework, comprising linear and non-linear ones. However, only the 15 ES methods with multiplicative error structures (heteroskedastic) were herein considered for TS analysis [21]–[32]. Not only do multiplicative error structures are conservative but they also yield more realistic 95% prediction interval (PI) values. Furthermore, a reduction in the number of ES methods evaluated also diminishes the expensive computational time.

The versatile and fully automatic ETS framework requires neither stationarity nor “strict” linearity to produce contemporaneous TS forecasts for variable time horizons (*h*) [21]–[32]. Consequently, it is extensively employed in, e.g., econometrics and inventory control where automatic forecasts are required for a large number of diverse TS. This forecasting framework, whose performance was recently and favorably compared to those of several forecasting techniques across thousands of TS [32], adapts to underlying alterations in disease dynamics and automatically revises forecasts *on-line* as new observations accumulate (Fig. 3). This adaptability is essential for epidemiological forecasting methods because interventions (e.g. medical and prophylactic treatment) almost ubiquitously perturb disease TS dynamics. An intuitive description of the ETS framework appears in Fig. 3; it is only succinctly described below because it has been meticulously derived elsewhere [21]–[31].

In the ETS framework, the expected mean of a forecasted observation, *E*[*F*(*y_t_*_+*h*_|*I_t_*)], is conditioned on the information set (*I_t_*) available at time *t*—i.e. these are external predictions. The information set *I_t_* contains unobserved level (*l_t_*), trend (*r_t_*), and or seasonal (*s_m|t_*: month, m = [1, 12]) components, whichever pertinent, depending on the underlying ES method. Possible lower-frequency “harmonics”, i.e. inter-annual fluctuations, are handled by *l_t_* and *r_t_* components in the ETS framework because the limited temporal window (01/1996–06/2004) considered in this investigation precludes stable estimation of periodicity much longer than 12 months. The observed amalgamated TS is symbolized by {*y_t_*}, as previously defined, whereas unobserved TS components enter the vector *x_t_*, according to the general state and transition Equations 2 & 3, respectively:
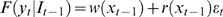
(2)

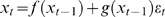
(3)where,
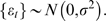



For ES methods with multiplicative error structures, *w*(*x_t_*_-1_) and *r*(*x_t_*_-1_) have both the form of the expected mean of a forecasted observation, *E*[*F*(*y_t_*|*I_t_*_-1_)]. Otherwise, *w*(*x_t_*_-1_) = *E*[*F*(*y_t_*|*I_t_*_-1_)] and *r*(*x_t_*_-1_) = 1 for ES methods with additive error structures (not discussed hereafter). All ES methods rely on the adjustment of *l_t_*, *r_t_*, and or *s_m|t_* TS components with their corresponding smoothing control *α*, *β*, and *γ* values; furthermore, *ϕ* controls smoothing of *r_t_*-dampening if present. Basically, contemporaneous forecasts obtain *via* TS extrapolations whereby previous deviations between forecasts and their corresponding observations are exponentially adjusted with *α*, *β*, *γ*, and or *ϕ*. Large smoothing control values confer greater weights to recent information and effectively shorten the smoothing “memory”, i.e. the recent-past has a more pronounced influence on estimated components than does the distant-past [11], [21]–[31]. Three important remarks: 1) a single or multiple smoothing control values may be required depending on which TS components are present in the selected ES method; 2) although smoothing controls are symbolized with the same notation across distinct ES methods, their function may vary from one ES method to another because the relationship between TS components may also differ (e.g. multiplicative *vs*. additive *r_t_*); last, 3) the function of smoothing control values approximately parallels that of the bandwidth in a one-side Nadaraya-Watson exponential kernel.

Smoothing controls plus unobserved components are estimated for all ES methods within the ETS state-space framework using a maximum likelihood function analog [31]. Here, the general ETS constraints are: 0<*α*, *ϕ*<1; 0<*β*<*α*; and, 0<*γ*<1−*α*; strictly multiplicative error structures; multiplicative *s_m|t_* values add annually to 12 because m = [1, 12]; and, 36 months [≡3*p*] for initial training, the possible specification of longer intervals notwithstanding. Defaulted ETS constraints are specified for several reasons [21]–[31] among them to prevent the forecast execution program from producing unrealistic results.

Once each ES method within the ETS framework is optimized at time *t*, that which minimizes the Akaike's Information Criterion (*AIC*) is selected to generate the *h*-month horizon forecast path distribution. The *h*-month horizon forecast path distribution, *F*(*y_t_*_+*h*_|*I_t_*), obtains *via* recursive iterations (Eqs. 2 & 3) of *B* = 1000 ordinary {*ε_t_*} bootstrap-generated pseudo-TS [11],[31],[33]. With the accumulation of each new observation, ES methods within the ETS framework are re-optimized and the best-performing ES method is re-selected based on the *AIC*. Subsequently, *F*(*y_t_*_+*h*_|*I_t_*) is again recursively generated from *B* = 1000 ordinary {*ε_t_*} bootstrap-generated pseudo-TS. For example, observations from January 1996 to December 1998 initialize the ETS execution program (Fig. 3) that predicts consultation rates for January 1999 to May 1999, assuming *h* = [1, 5]. Once the January 1999 forecast plus the available TS (including the most contemporaneous observation of January 1999) become available to the *on-line* system (Fig. 3), the execution program cycles again and optimizes all considered ES methods, re-selecting the best-performing one (which may or may not be the same one employed prior to the arrival of the new observation). As a result, revised consultation rate predictions for February 1999 to June 1999 ensue. This process repeats *ad infinitum* (Fig. 3). The 95% PI values for the simulated *F*(*y_t_*_+*h*_|*I_t_*) paths are estimated from distribution percentiles.

Although a full portrayal of the ETS framework (Eqs. 2 & 3) encapsulating the 15 considered ES methods [21]–[31] is beyond the scope of this investigation, those ES methods which have been selected at least once during this TS analysis are described in terms of *E*[*F*(*y_t_*|*I_t_*_-1_)] and *x_t_* recursions (**Table 2**). [Table 2 caption also provides an ES method example explicitly written in matrix notation.] As discussed afterwards in the *Results* section, none of the selected ES methods (Table 2) is seasonal, reflecting the endemicity of the TS analyzed herein. For further details concerning the ETS framework, refer to, e.g., Hyndman *et al.* [27],[29],[31].

**Table 2:** Selected exponential smoothing methods within the state-space forecasting framework.

ETS	*E*[*F*(*y_t_*|*I_t-1_*)]	*x_t_*
**MNN**	*E*[*F*(*y_t_*|*I_t_*_-1_)] = *l_t_*_-1_	*l_t_* = *l_t_*_-1_(1+*αε_t_*)
**MA_d_N**	*E*[*F*(*y_t_*|*I_t_*_-1_)] = *l_t_*_-1_+*ϕb_t_*_-1_	*l_t_* = (*l_t_*_-1_+*ϕb_t_*_-1_)(1+*αε_t_*)
		*b_t_*_-1_ = *ϕb_t_*_-1_+*β*(*l_t_*_-1_+*ϕb_t_*_-1_)*ε_t_*
**MM_d_N**	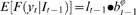	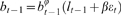

All exponential smoothing (ES) methods within the state-space forecasting (ETS) framework (Equations 2 & 3) were optimized with a likelihood function analog as new *Schistosoma haematobium* time-series (TS) observations for the district of Niono, Mali, became available; the best-performing method was continuously re-selected with the Akaike's Information Criterion (*AIC*) to generate optimum forecasts (*Methods*). Throughout the investigational period, only 3 from a total of 15 ES methods considered within the ETS framework were re-selected; they are: the multiplicative error/ trendless/ aseasonal (MNN); multiplicative error/ damped additive trend/ aseasonal (MA_d_N); and, multiplicative error/ damped multiplicative trend/ aseasonal (MM_d_N) ES methods. Notice that none of them are seasonal. Although a full portrayal of the ETS state-space framework (Equations 2 & 3) encapsulating all 30 ES methods [11], [21]–[31] is beyond the scope of this investigation, those ES methods which have been selected at least once during the TS analysis are described herein in terms of *E*[*F*(*y_t_*|*I_t_*_-1_)] and *x_t_* recursions—*α*, *β* , and *ϕ* control smoothing of level (*l_t_*), trend (*r_t_*), and *r_t_*-dampening, respectively. Large *α*, *β*, and *ϕ* values confer greater weights to recent information and effectively shorten the smoothing “memory”, i.e. the recent-past has a more pronounced influence on estimated components than does the distant-past [11], [21]–[31]. For example, MA_d_N state-space Eqs. 2 & 3 may be written in explicit matrix form as: *F*(*y_t_*|*I_t_*_-1_) = A•*x_t_*_-1_•(1+*ε_t_*) & *x_t_* = B•*x_t_*_-1_+A•*x_t_*•C•*ε_t_* where A = (1, *ϕ*)′, *x_t_*_-1_ = (*l_t_*_-1_, *r_t_*_-1_), C = (*α*, *β*), and B is a 2×2 matrix whose entries b_1,1_, b_1,2_, b_2,1_, b_2,2_ are 1, *ϕ*, 0, *ϕ*, respectively.

### Forecasting accuracy and dispersion

Standard accuracy and dispersion measures were employed in this analysis. Accuracy—which measures the forecasting competence—is defined here as the mean absolute percentage error (*MAPE*) between observed and forecasted TS values whilst infrequently reported PI values reflect the dispersion of forecast distributions; the dispersion of simulated *F*(*y_t_*_+*h*_|*I_t_*) probability density functions were also summarized as the average coefficient of variance (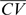). *MAPE* and 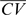 values are calculated with Equations 4 & 5, respectively:
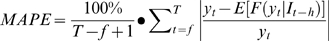
(4)

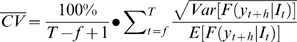
(5)

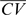
 and *MAPE* (external) values are expressed in percentage (%) as a function of the *h*-month horizon forecast. In Eqs. 4 & 5, *T* is the TS length and *f* = 3*p*−1+*h* reflects the actual time when the *h*-month horizon forecast begins. Large *MAPE* and 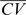 values imply low accuracy and large dispersion, respectively, and *vice-versa*. The distinction between *MAPE* and PI (or 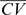) values is an important one. The first assesses the competence, i.e. the skill, of the *h*-month horizon forecast; the latter only measures the dispersion of the *h*-month horizon forecast path distribution. Thus, PI (or 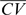) values have paramount importance for calculating, e.g., the probability that a future observation will be smaller or greater than the expected forecast distribution mean by a certain margin. Likewise, the number of individuals at risk may be calculated for a specified probability.

This TS has not undergone Box-Cox transformations. Notice however, that TS frequently undergo such transformations prior to the forecasting analysis. Regardless, contemporaneous forecasts and standard accuracy measures (e.g. *MAPE*) must be (and were) superimposed onto and computed for, respectively, the originally observed TS because accuracy may be severely distorted in the transformed dimension—i.e. occasionally, forecasts may be simultaneously accurate and inaccurate in the transformed and original dimensions, respectively. All calculations were performed in R: A language and environment for statistical computing [30],[31].

## Results

**Figure 4:**
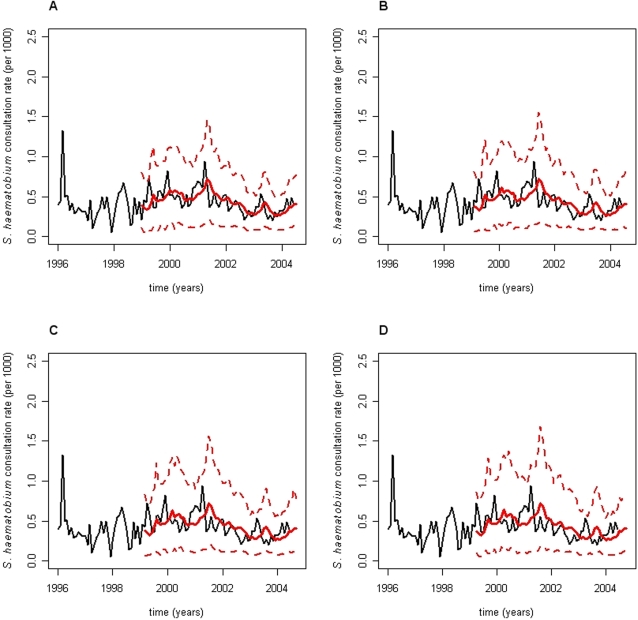
State-space forecasts of *Schistosoma haematobium* consultation rate time-series for the district of Niono, Mali. Observed *Schistosoma haematobium* consultation rate time-series (TS) in the district of Niono, Mali, are depicted as black lines in this composite panel while red traces correspond to contemporaneous *h*-month horizon forecasts; 95% prediction interval (PI) bounds are symbolized by red dots of the same color. Abscissa projections span 102 months (01/1996–06/2004) while ordinate scales represent the number of newly diagnosed (or forecasted) *S. haematobium*–induced terminal hematuria cases *per* 1000 individuals. Forecasts were generated with exponential smoothing (ES) methods, which are encapsulated within the state-space forecasting (ETS) framework (*Methods*). Panels A, B, C, and D correspond to 2-, 3-, 4-, and 5-month horizon forecasts, respectively. These forecasts are, by definition, external predictions. Predictions were superimposed onto the original TS to allow visual prediction accuracy evaluation. This figure should be considered dynamically. As observations and forecasts became available to and from the *on-line* execution program, the actual graphing of forecasts (red traces) preceded that of observations (black lines) by exactly *h*-month horizon.

This longitudinal retrospective (01/1996–06/2004) investigation analyzed the *S. haematobium* consultation rate TS for the district of Niono, Mali. In **Figure 4**, the observed amalgamated *S. haematobium* consultation rate TS is symbolized by black lines. The TS is excessively noisy from 1996 to 1999 when a sharp rise in consultation rates clearly ensues. From 2001 onwards, consultation rates decline because of large-scale prophylactic de-parasitation programs. Regardless, 2- to 5-month horizon forecasts clearly captured these inter-annual tendencies (Fig. 4)—red traces correspond to contemporaneous *on-line* 2-, 3-, 4-, and 5-month horizon forecasts (panels A, B, C, and D, respectively) whilst their 95% PI values are depicted in dots of the same color. Abscissa TS projections span 102 months (01/1996–06/2004) while ordinate scales represent the number of newly diagnosed (or forecasted) *S. haematobium*–induced terminal hematuria cases *per* 1000 individuals.

TS observations were continuously submitted to a family of general-purpose ES methods—encapsulated within the ETS state-space framework—to produce contemporaneous *on-line* forecasts (i.e. external predictions). Predictions were superimposed onto the original TS to allow visual evaluation of prediction accuracy. While this superimposition is absolutely essential, it might convey the false impression that forecasts represent internal predictions—this is not the case. Fig. 4 should be considered dynamically. As observations and forecasts become available to and from the *on-line* forecast execution program (Fig. 3), respectively, the actual graphing of forecasts (red traces) precede that of observations (black lines) by exactly *h*-month horizon.

Generally, the ETS framework accommodates seasonal and inter-annual fluctuations, producing reasonably accurate TS forecasts. Here, inter-annual fluctuations dominate the *S. haematobium* TS while seasonal oscillations are practically inexistent (Fig. 4). These fluctuations are intuited from the observed consultation rate TS (black lines), as well as implied by the absence of {*s_t_*_|*m*_} *vis-à-vis* the presence of {*l_t_*} and or {*r_t_*} components in automatically selected ES methods (Table 2). Only 3 ES methods were automatically selected with the *AIC* during this *S. haematobium* TS forecasting analysis. These selected ES methods, which have been described in terms of *E*[*F*(*y_t_*|*I_t_*_-1_)] and *x_t_* recursions (Table 2), are: the multiplicative error/ trendless/ aseasonal (MNN), multiplicative error/ damped additive trend/ aseasonal (MA_d_N), and multiplicative error/ damped multiplicative trend/ aseasonal (MM_d_N) ES methods. None of them are seasonal and hence exogenous forcing (e.g. climate covariates) was not invoked to improve predictions.

**Table 3** lists the frequency (*n*) with which these ES methods were re-selected during the forecasted investigational period plus the method-specific median (and IQR: inter-quartile range) of pertinent smoothing control values. Smoothing control values are time-dependent because they are continuously re-estimated as new observations accumulate. Yet, their magnitude drifts little in this investigation. Hence, they were reported as median and IQR values. The MNN smoothing control *α* is obviously large since this method only has a level {*l_t_*} component, i.e. the MNN ES method lacks {*r_t_*} and {*s_t_*_|*m*_} components as well as their corresponding smoothing control *β*, *ϕ* and *γ* values. For MA_d_N and MM_d_N methods, *β*≤*α*≪*ϕ* due to large dampening of minute *r_t_* components. As new observations accumulated, the automatic and criterial re-selection of ES methods conferred an additional layer of flexibility to the ETS framework and consequent TS forecasts. [Smoothing control values may differ in functional form across ES methods despite the retained notation (*Methods*).]

**Table 3:** ETS framework smoothing controls.

ETS	*n*	*α*	*β*	*ϕ*
		**Median (IQR)**	**Median (IQR)**	**Median (IQR)**
**MNN**	45	0.35 (0.04)	-	-
**MA_d_N**	6	0.05 (0.03)	0.05 (<0.01)	0.80 (0.01)
**MM_d_N**	16	0.08 (0.03)	0.03 (0.03)	0.82 (0.01)

Three exponential smoothing (ES) methods within the state-space forecasting (ETS) framework employed herein were automatically selected *n* times each, according to the Akaike's Information Criterion (*AIC*), to forecast *Schistosoma haematobium*–induced terminal hematuria consultation rate time-series (TS) for the district of Niono, Mali (1996–2004). The multiplicative error/ trendless/ aseasonal (MNN), multiplicative error/ damped additive trend/ aseasonal (MA_d_N), and multiplicative error/ damped multiplicative trend/ aseasonal (MM_d_N) ES methods were selected 45, 6, and 16 times, respectively. Though the estimated smoothing controls for each of these ES method are time-dependent, they fluctuate only slightly (*Results*). Thus, they are reported above as median and inter-quartile range (IQR) values. These three methods are remarkably similar. The MNN was the most frequently selected ES method (*n* = 45). Only the *α* value was listed for this method because it only has a level (*l_t_*) TS component; *β* and *ϕ*, are reserved for methods that have trend (*r_t_*) and *r_t_*-dampening TS components (i.e. MA_d_N and MM_d_N). For MA_d_N and MM_d_N methods, *β*≤*α*≪*ϕ* due to large dampening of minute *r_t_* TS components. Of note, smoothing controls differ in function across ES methods, their retained notation notwithstanding. In sum, only aseasonal methods with minute or inexistent *r_t_* plus significant *l_t_* TS components were automatically selected during the investigational period, suggesting that: 1) TS forcing by seasonal covariates is not limiting; and, 2) public health intervention, population behavior, migration, and irrigation management may govern *S. haematobium*–induced terminal hematuria consultation rate TS fluctuations in this district. The strength of the forecasting approach employed herein relies on the automatic and systematic *AIC*-directed switches between ES methods within the ETS framework as new observations accumulate, conferring an additional layer of flexibility to TS predictions.

**Figure 5:**
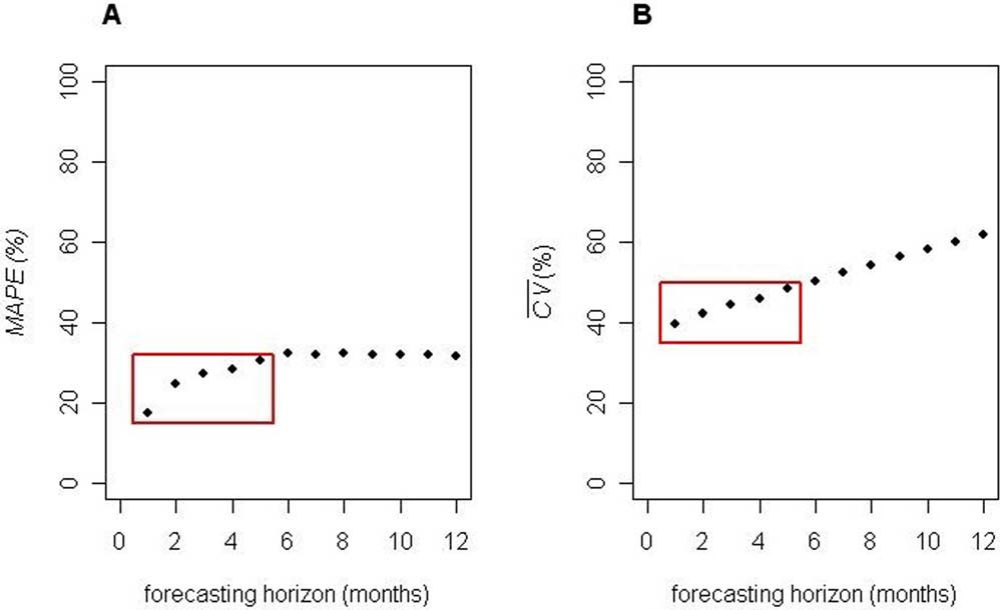
*Schistosoma haematobium* consultation rate time-series forecasting accuracy and dispersion for the district of Niono, Mali. Panel A: Mean absolute percentage error (*MAPE*) values between *Schistosoma haematobium* time-series (TS) observations for the district of Niono, Mali, and their corresponding *h*-month horizon forecasts measure external accuracy. The average coefficient of variance (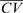) for *h*-month horizon forecast probability density functions reflect prediction dispersion. *MAPE* and 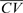 values are displayed as a function of *h*-month horizon forecasts. *MAPE* and 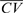 values for 1–5 month horizon forecasts were *circa* 25 and 45%, respectively. Therefore, panels A and B demonstrate that forecast accuracy and dispersion are reasonable for short horizons. Of note, *MAPE*, unlike 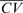, values assess the skill of *h*-month horizon forecasts. 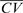 and PI values are rarely reported outside the econometric literature; yet, they have paramount importance for calculating, e.g., the probability that a future observation will be smaller or greater than the expected forecast distribution mean by a certain margin. Alternatively, the number of individuals at risk may be calculated for a specified probability.

*MAPE* and 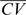 values for 1- to 5-month horizon forecasts were *circa* 25 and 45%, respectively (**Figure 5**). 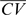 values reflect the average dispersion of simulated *F*(*y_t_*_+*h*_|*I_t_*) probability density functions whilst *MAPE* values measure the mean absolute percentage error between TS observations and their forecasts. Accuracy (*MAPE*; panel A) becomes approximately asymptotic as the *h*-month horizon forecast increases beyond 6 months because of a minute {*r_t_*} component irrespectively of the selected ES method, significant inter-annual {*l_t_*} fluctuations notwithstanding. As expected, dispersion (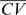; panel B) increases as innovations propagate through longer stochastic *h*-month horizon forecast paths.

## Discussion

*Schistosoma* sp. expose and infect more than 500 and 200 million individuals in 77 countries, respectively. In the Sahel, *S. haematobium* is endemic and highly prevalent [2], [10]–[15]. The few reports evaluating *S. haematobium* transmission in Mali [10]–[15], particularly in the district of Niono (Fig. 1), suggest that forecasting *S. haematobium* consultation rate TS may locally assist with reducing morbidity. For instance, *S. haematobium* is the 5^th^ most frequently diagnosed infection (the 6^th^ commonest consultation etiology); it accounts for 2.5% of total CSCOM service area consultations [11],[20] with 50 to 75% community prevalence [12],[13] in the district of Niono. Paradoxically, temporal *S. haematobium* analyses are scarcely reported in the parasitic literature e.g. [16]–[18] probably because 1) this neglected tropical disease is endemic whereas most infectious disease TS forecasts usually attempt to detect epidemics, i.e. unexpected rises in consultation rate first moments, assisting with tailoring control measures; 2) *S. haematobium* TS tend to be excessively noisy, hindering analyses; finally, 3) long delays between *S. haematobium* infection and diagnosis challenge efforts to relate predicted high consultation rates to their potentially preventable sources. Notice that, though endemic, *S. haematobium* TS does fluctuate.

The ETS framework employed herein reasonably forecasted long horizons (Fig. 4), partially circumventing the limitations imposed by the *S. haematobium* TS noisy level and long delays between infection and diagnosis. Thus, this report addresses challenges in, and the scarcity of, *S. haematobium* TS forecasting reports with the flexible ETS framework (Fig. 3), which may locally assist with managing endemic *S. haematobium* transmission in the district of Niono, Mali. Here, accuracy (i.e. *MAPE*) and dispersion (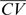) for contemporaneous (“out-of-fit”) 1- to 5-month horizon *S. haematobium* consultation rate TS forecasts were *circa* 25 and 45%, respectively (Figs. 5). *MAPE* values assess the competence, i.e. the skill, of *h*-month horizon forecasts; 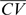 (or PI) values measure the dispersion of *h*-month horizon forecast path distributions. The later has paramount importance for calculating, e.g., the probability that a future observation will be smaller or greater than the expected forecast distribution mean by a certain margin. Likewise, the number of individuals at risk may be calculated for a specified probability. The rarely considered 2^nd^ moment forecasts (PI) may significantly assist authorities with risk and scenario analyses.

A comprehensive *S. haematobium* intervention strategy depends not only on prevalence, which has already been discussed in the *Introduction* section [10]–[15], but also on incidence measures. For instance, an abnormal rise in incidence should alarm authorities who are charged with investigating and containing hazard, ensuring that CSCOM service areas are able to handle patient demand, sensitize communities, control transmission, and monitor intervention impact. Thus, it is important to delineate some parallels between the *S. haematobium* consultation rate TS plus their forecasts analyzed herein (Fig. 4) and the unobserved incidence.

The monthly *S. haematobium* consultation rate is proportional to the unobserved monthly incidence TS—i.e. an increase in the monthly *S. haematobium* consultation rate most likely stems from a rise in the monthly incidence TS since the former is a fraction of the latter. The observed and forecasted consultation rate TS (Fig. 4) approximately reflect the monthly *S. haematobium*-induced terminal hematuria incidence because ∼95% of the Niono district population lives within 15 km of CSCOM facilities and hematuria alarmingly prompts patients to access available treatment. Although these records [19],[20] are unsuitable for estimating the exact *S. haematobium* incidence, it may be approximated to at least an order of magnitude higher than the observed consultation rate TS under mean-field, steady-state, stable age structure, constant population growth (3.2%), and overall prevalence (∼60%) assumptions. Consequently, the difference between the observed consultation rate (Fig. 4) and the estimated incidence TS described above (not shown) approximately reflects the *S. haematobium* incidence of asymptomatic and mildly symptomatic cases. The effective *S. haematobium* incidence depends on age as recurrent cercarial exposure induces partial-immunity [1]. *S. haematobium*-induced terminal hematuria consultations emanate primarily from the 7–14 age-category, which comprises 20 to 30% of the district population [11],[19],[20]. Thus, a rough population structure TS adjustment suggests that the actual and forecasted *S. haematobium*-induced terminal hematuria incidence is roughly 3 to 5 times higher in the aforementioned age-category.

The dependency of *S. haematobium* transmission on the environment is extremely important and cannot be understated. *S. haematobium* transmission depends on climate [1],[18], as well as natural (e.g. lakes) and artificial (e.g. irrigation schemes) water reservoirs [1],[2]. Despite these dependencies, covariates such as climate were not invoked to forecast the *S. haematobium* TS because it is endemic [10]–[13] and aseasonal (Fig. 4 and Tables 2 & 3) in the district of Niono, Mali. In this district, temperature and rainfall TS values guarantee *S. haematobium* transmission suitability throughout the year—i.e. transmission is not limited here by climate thresholds beyond which the *S. haematobium* life-cycle becomes unstable.

Unlike temperature, rainfall TS values exhibit large (inter-tropical convergence zone-mediated) inter-annual oscillations in the Sahel. These fluctuations prompt the local authority (*Office du Niger*) to accordingly adjust irrigation management, which inevitably and transiently alters *S. haematobium* transmission suitability in this district. In other words, rainfall precipitation only indirectly affects *S. haematobium* transmission in this district. For example, an augment in rainfall precipitation increases water availability. Consequently, the *Office du Niger* may relax water control to better irrigate drier areas while collaterally enhancing water-flow through typically well-served agricultural fields—*S. haematobium* transmission suitability could then simultaneously increase and decrease in the former and latter scenarios, respectively. Another major source of TS fluctuations stems from the constant exposure to, and behavioral risks associated with, the irrigation system (Fig. 2). These TS fluctuations are further aggravated by the influx of migrant workers from non-endemic areas. The variable clinical course of *S. haematobium*-induced terminal hematuria may also introduce stochastic fluctuations into this TS. Finally, the impact of large-scale prophylactic de-parasitation programs perturbs *S. haematobium* transmission as evidenced by sustained consultation rate declines from 2001 onwards (Fig. 4). Consequently, *S. haematobium* TS fluctuations in this district require forecasts, the endemecity of this neglected tropical disease notwithstanding.

Future studies should dedicatedly investigate the intricate roles of geography, climate, irrigation management, and human behavior (including migration) in the context of *S. haematobium* transmission ecology to improve forecasts and interventions in this district. Unfortunately, addressing the multidimensionality of this disease remains difficult owing to poor documentation. Until this information becomes available, the employment of univariate methods (e.g. ETS framework) to forecast *S. haematobium*-induced terminal hematuria incidence in the district of Niono seems adequate. This is consistent, for example, with the successful employment of univariate methods to forecast schistosomiasis TS in Dongting Lake, China [16], albeit with the admonition that these results cannot be indiscriminately generalized to any location.

Furthermore, this *S. haematobium*-induced terminal hematuria TS is aseasonal (Fig. 4 and Tables 2 & 3), which intuitively argues against the incorporation of seasonal climate covariates and corroborates the employment of univariate prediction methods. [The automatically selected MNN, MA_d_N, and MM_d_N forecasting methods (Tables 2 & 3) are very similar; they reflect the fact that the *S. haematobium*-induced terminal hematuria TS is aseasonal, quasi-trendless, with significant inter-annual fluctuations in the district of Niono, Mali.] *S. haematobium* transmission generally drifts slowly in response to also slow climate and or other environmental changes. As a result, the ETS framework has the benefit of time to adapt to perturbations in and revise forecasts for this fully-stable (endemic) *S. haematobium* TS. In other words, current observations mirror past disease dynamics and environmental interactions. Forecasting methods that capture these relationships through historical TS analysis often reflect prior and present interactions on post-sample (external) predictions. This is clearly not the case when the chaotic weather or a newly erected dam, for example, suddenly inundate large areas triggering outbreaks and epidemics (i.e. under unstable transmission conditions). While it is difficult to predict weather, environmental impact may be investigated with satellite technology; for example, Beck-Wörner *et al.* [34] successfully employed a hybrid of remotely-sensed and surveyed data from western Côte d'Ivoire to spatially predict *S. mansoni* infection risk. [Although the consultation records analyzed herein are resolved by 17 CSCOM service areas, spatial considerations were dismissed because the district of Niono occupies only ∼20 000 km^2^ (Fig. 1).]

Conversely, lagged weather- and or climate-based models are particularly powerful whenever disease transmission is unstable and epidemics are suddenly-triggered. For example, a weather-based Poisson regression (4^th^-degree polynomial distributed lag) was employed to model malaria TS in highly unstable regions of Ethiopia [35]. However, lagged weather- and or climate-based models not only demand extensive programming and expertise to reasonably specify the number of lags but they also require caution to avoid multicollinearity, problematic optimization, and lengthy TS requirements. Furthermore, lagged models, unlike ES methods, must be tailored to each disease because the optimum functional form of climate covariates is not obvious [35]–[38]. Weather events must be measured because predicting its chaotic nature with several weeks in advance is usually impossible. Predicting climate is not trivial and such predictions are typically too global to substantially add local forecasting accuracy. Otherwise, weather- and or climate-based models are crucial to: elucidate complex disease transmission behavior [37], forecast long horizons [38], and model infectious disease transmission in the spatial dimension [18],[36]. If the optimum functional form of climate covariates is unveiled [37] then reasonable forecasts yield [38]. While some form of lagged weather- and or climate-based model may be indispensable in certain cases [35]–[38], simpler ES alternatives may locally forecast fully- and or partially-stable disease TS, e.g. *meso*-endemic malaria [11] and endemic *S. haematobium* transmission in the district of Niono, Mali.

Like other forecasting approaches, ES methods perform reasonably well whenever disease transmission comprises relatively large event-probabilities during long investigational periods. Forecasting methods surrender when disease transmission depends on rare stochastic events (in highly-structured populations), each associated with minute (albeit finite) probabilities, governing unstable and transient disease dynamics. These highly-stochastic structured disease dynamics feature sudden epidemic resurgence and ample epidemic volume variability that are not easily investigated with univariate and most multivariate methods, often requiring more sophisticated approaches e.g. [39],[40].

The generality, reasonable performance, and operational simplicity of the ETS forecasting framework employed herein may appeal to those working towards infectious disease hazard mitigation. Computationally, recursive ES methods (Table 2), encapsulated within this framework, may be easily and automatically optimized, as well as operated, by non-statisticians in the public health sector [21]–[32]. They are often available as software procedures (e.g. SPSS and EViews), pre-written functions for programming environments (e.g. S-plus and the freely-available R language and environment for statistical computing), and scripts in classical programming languages (e.g. FORTRAN and C). This has been previously discussed in Medina *et al.* [11].

Moreover, ES methods adapt with an *on-line* training (Fig. 3) that exponentially discounts prior information, i.e. information from the recent-past is more relevant to forecasts than those from the distant-past. Its versatility reflects “density-estimation” of unobserved TS components (*Methods*) [41]. Owing to both adaptability and versatility, ES methods tend to accommodate intervention-induced perturbations (e.g., medical and prophylactic treatment) that inherently plague longitudinal retrospective disease TS investigations (Fig. 4) e.g. [3]–[7] as well as disease TS with distinct transmission modes [11], respectively. This is illustrated here by the *S. haematobium* monthly consultation rate declines from 2001 onwards (owing to large-scale prophylactic de-parasitation programs) and corresponding 2- to 5-month horizon TS forecasts, which captured these inter-annual tendencies (Fig. 4).

Forecasting and early warning systems for managing infectious diseases depend on human behavior, population, disease TS, climate, environment and or a combination thereof, whichever alternative best compromises among realism, feasibility, robustness, and parsimony. Nevertheless, forecasts do not obligatorily require exogenous covariates. Medina *et al.* [11] demonstrated how a robust univariate general-purpose ES method may produce contemporaneous (“out-of-fit”) forecasts for dissimilar diseases without disease-specific tailoring of the forecasting method. More recently, Chaves & Pascual demonstrated the importance of assessing the performance of several forecasting methods, including climate-based ones, in a systematic fashion [38]. Finally, the aforementioned ideas were successfully combined here to allow *AIC*-directed switches (as new TS observations accumulated and perturbations evolved) among 15 general-purpose ES methods within the ETS framework, further improving forecasts (Fig. 4 & 5 and Tables 2 & 3).

Sudden TS perturbations transiently limit the performance of this and other forecasting approaches. Like most forecasting approaches, particularly univariate ones, ES methods react only after initial TS fluctuations ensue. Thus, this limitation is not unique to ES methods employed herein. Introducing covariates may lessen this limitation if, and only if, the underlying covariate fluctuation is either measurable or predictable—this is often, but not always, the case. Furthermore, the deleterious effects of sudden, even if small, TS perturbations propagate through *h*-month horizon forecast paths. This phenomenon clearly surfaced in Fig. 4 (panels A, B, C, and D). As the horizon increased from 2- to 5-month, forecasts became progressively worse (Fig. 5) for sudden consultation rate TS fluctuations in 2001 (Fig. 4) as previously discussed.

A major limitation of all TS analyses, and this investigation is not exempt from it, consists of information unavailability. The intricate role of geography, rainfall, irrigation management, and human behavior (including migration) in the *S. haematobium* transmission ecology has not been extensively documented for this district. Thus, general, adaptable, and versatile univariate ES methods were employed herein to generate forecasts. Second, missing monthly consultation records could have potentially introduced bias in this monthly *S. haematobium* consultation rate TS. However, this is unlikely owing to the random distribution of missing records across CSCOM service areas, months, and years. As listed in Table 1, missing records distribute approximately normally across CSCOM service areas and approximately uniformly through the investigational period [11]. The percentage of missing monthly records in the amalgamated TS is *circa* 17%, generally less than 2% *per* year. The only exception manifests in the practically reconstructed year of 1997 that was employed for program initialization—nevertheless, this is minimally consequential because program initialization would otherwise reflect the customary (and arbitrary) “opinion of an expert” [11].

### Conclusion

Changes in multiple dimensions (e.g. human behavior, population, disease TS, climate, and environment) will confer an ever-increasing role to infectious diseases forecasting and early warning systems. These predictive systems are based upon a single dimension or a combination thereof, whichever alternative best compromises among realism, feasibility, robustness, and parsimony. With the mounting evidence that *S. haematobium*—a neglected tropical disease—imposes an enormous burden on developing countries, public health programs therein could benefit from parsimonious forecasting and early warning systems to enhance management and control of this parasitic infection. Not only does this report address the paucity of *S. haematobium* TS forecasting investigations but it also advocates the usage of parsimonious state-space frameworks to forecast neglected tropical diseases. The ETS state-space forecasting framework employed herein generated reasonable 1- to 5-month horizon *S. haematobium* TS forecasts, obliquely capturing prior non-linear interactions between disease dynamics and exogenous covariates (e.g. climate) and hence, obviating the need for more complex predictive methods in the district of Niono, Mali. Thus, this and other e.g. [11], [21]–[32] results suggest that the remarkable performance of state-space forecasting methods since the 1960s may be capitalized by the public health sector, providing a basis for local re-organization and strengthening of intervention programs in this and potentially other Sahelian districts. The operational simplicity, generality, and flexibility of state-space frameworks, such as the one employed here, conveniently allow for: 1) unsupervised model selection without disease-specific methodological tailoring; 2) *on-line* adaptation to fluctuations in partially- and fully-stable disease TS; and, 3) automatic switches between distinct forecasting methods as new TS perturbations dictate. Generally, state-space approaches are malleable to the dynamic incorporation of covariates (e.g. climate), expert opinion, and even a spatial dimension as needed. Therefore, fully automatic and user-friendly state-space forecasting frameworks, incorporating myriad (e.g. univariate, multivariate, and spatial-temporal) options, could considerably enhance disease control and hazard mitigation in regions where vulnerability to neglected tropical diseases is pervasive and statistical expertise is scarce.
